# Mid-aortic syndrome in two Saudi children with refractory hypertension: a case report

**DOI:** 10.1186/s43044-026-00758-z

**Published:** 2026-06-24

**Authors:** Osama Alrusayni, Yazeed Saleh Alahmed, Yasser Ahmad Bhat, Raghad Abdulaziz Alhojailan, Raghad Husain Alkhalifah, Insha Banday, Fahad Alhabshan

**Affiliations:** 1 Pediatric Cardiology Department, Prince Sultan Cardiac Center-Qassim, Qassim Health Cluster, MOH, Qassim, Saudi Arabia; 2 Department of Pediatrics, College of Medicine, Qassim University, Qassim, Saudi Arabia; 3 Department of Cardiology, Medical City, , Qassim University, Qassim, Saudi Arabia; 4 College of Medicine, Qassim University, Qassim, Saudi Arabia; 5 Maternity and Children Hospital, Qassim, Saudi Arabia; 6 Paediatric Cardiology Department, King Abdulaziz Cardiac Center, Ministry of National Guard, National Guard Health Affairs, Riyadh, Saudi Arabia

**Keywords:** Mid-aortic syndrome, Hypertension, Aortic coarctation, Aortic stenosis

## Abstract

**Background:**

Middle aortic syndrome (MAS) is a rare clinical condition in children that is associated with a significant risk of morbidity and mortality. Delayed diagnosis and inadequate treatment can lead to severe complications, resulting in life-threatening consequences.

**Case presentation:**

In this paper, we present two cases involving two 10-year-old Saudi girls diagnosed with MAS who presented with systemic hypertension. Echocardiography demonstrated a patent aortic arch; however, it showed abnormal diastolic flow in the descending aorta, which facilitated the diagnosis of MAS.

**Conclusions:**

Even when a paediatric patient shows no symptoms, health care workers should not record unreliable blood pressure readings. These blood pressure readings may indicate the onset of a disease that, if left untreated, could have catastrophic consequences. This report emphasises the rarity and clinical importance of MAS in the paediatric population, aiming to enhance early diagnosis and improve management strategies. Moreover, these findings underscore the critical role of echocardiography in diagnosing MAS.

**Supplementary Information:**

The online version contains supplementary material available at 10.1186/s43044-026-00758-z.

## Introduction

Coarctation of the aorta (CoA) typically occurs at the aortic isthmus, which is the segment of the thoracic aorta immediately following the left subclavian artery. Nevertheless, in rare instances, CoA may manifest in alternative regions of the aorta, a condition referred to as atypical CoA. One such example is middle aortic coarctation, or mid-aortic syndrome (MAS), in which the constriction occurs in the midsection of the aorta, frequently affecting the abdominal aorta and, in some cases, the renal arteries [1]. MAS constitutes approximately 0.5–2% of all cases of aortic narrowing, with clinical symptoms typically emerging before age 18. Hypertension (HTN) represents the predominant clinical presentation and may lead to severe complications such as hypertensive encephalopathy, stroke, and congestive heart failure. Additional clinical manifestations may include fatigue, failure to thrive, headache, nausea and/or vomiting, abdominal angina, and claudication [2].

In this report, we present two ten-year-old Saudi girls who were previously healthy but developed systemic hypertension, emphasising the need for thorough evaluation and high suspicion to avoid missed diagnoses of MAS.

### Patient 1

A 10-year-old female patient from Saudi Arabia who was previously in good health presented with a four-day history of dizziness and episodes of transient loss of consciousness, each lasting a few seconds. She regained consciousness spontaneously and was subsequently admitted to a local hospital, where she was found to exhibit significantly elevated blood pressure (BP), measured at 180/100 mmHg. The patient was managed under the diagnosis of hypertensive urgency and was referred to our specialised centre for further evaluation and treatment.

Upon examination, the patient did not present with dysmorphic features. Her vital signs were largely within normal limits, except for elevated blood pressure. The growth parameters included a weight of 34 kg, placing the patient at the 50th percentile; a height of 152 cm, corresponding to the 90th percentile; and a body mass index of 14.5 kg/m². A significant arm-leg blood pressure gradient of 52 mmHg was observed: the right upper arm blood pressure was 150/83 mmHg, the left upper arm pressure was 150/91 mmHg, the right leg pressure was 98/57 mmHg, and the left leg pressure was 98/69 mmHg. Pulses in the lower limbs were weak, whereas radial pulses were notably bounding. Cardiovascular assessment revealed normal first and second heart sounds and an ejection systolic murmur. The capillary refill time was less than 2 s. Other systemic examinations yielded unremarkable findings.

**Fig. 1:**
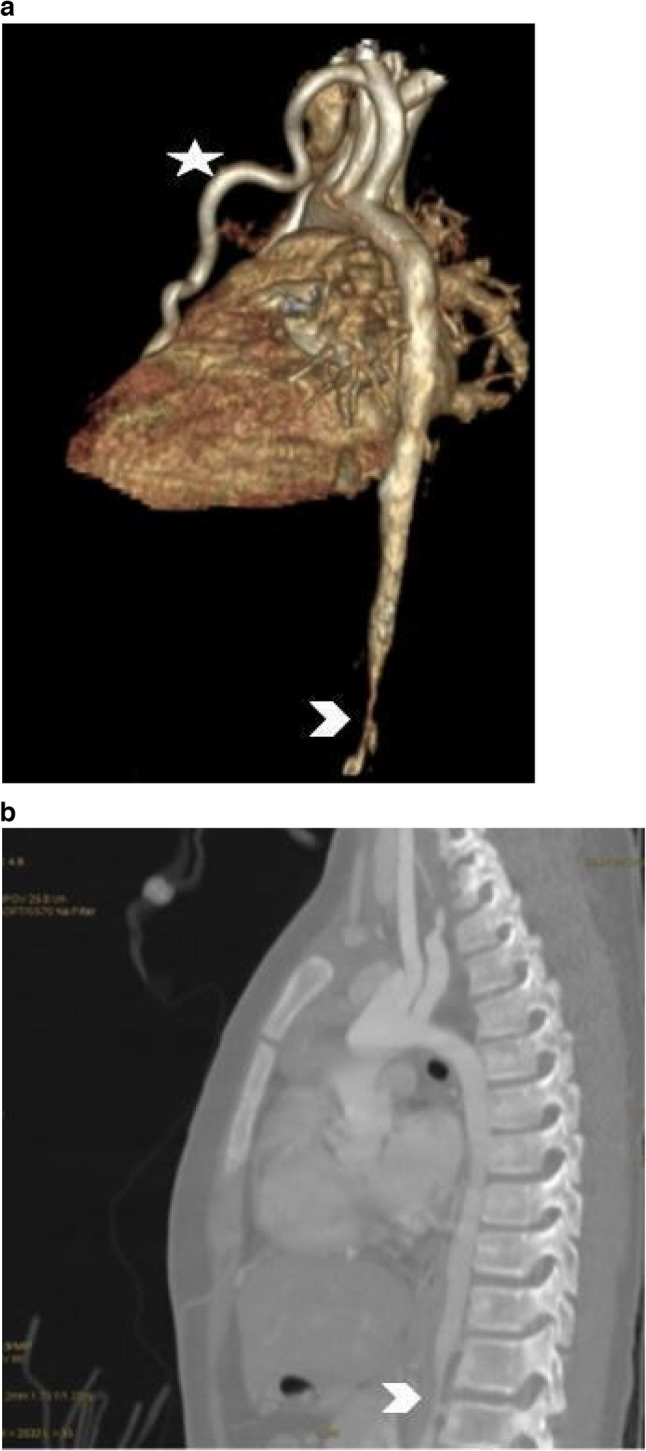
**A** CTA volumetric rendering revealed a dilated LIMA (star) and focal abdominal aortic narrowing (arrowhead). **B** The sagittal view revealed significant segmental narrowing (arrowhead) in the abdominal aorta

Initial investigations indicated that renal function was within normal limits. Findings from the echocardiogram revealed mild left ventricular hypertrophy (LVH) and a reduced calibre of the abdominal aorta. In addition, Spectral Doppler interrogation of the abdominal segment of the aorta showed constant forward diastolic flow, a delayed systolic upstroke, and continuous low-velocity flow. These observations raised concerns about potential obstruction at the level of the abdominal aorta, necessitating further evaluation with computed tomographic angiography (CTA) (Fig. 1a and b).

Renal Doppler assessment revealed low peak systolic velocities with a widened slope, indicative of pulsus parvus et tardus waveforms in both intrarenal arteries. This particular finding suggests the possibility of upstream vascular stenosis in the abdominal aorta. Notably, abdominal and pelvic ultrasound examinations were unremarkable.

Following the confirmation of the MAS diagnosis, the patient was referred to a specialised medical centre for further evaluation, where whole-exome sequencing was performed. The results of this analysis were inconclusive; however, an incidental finding revealed a homozygous variant of uncertain significance in the F12 gene. This gene is associated with F12 deficiency, which may occur if a pathognomonic mutation is inherited in an autosomal recessive manner. The medical team at the specialised centre determined that the patient would be managed conservatively with medication.

### Patient 2

A 10-year-old Saudi female patient who was previously in good health presented to a local medical facility with complaints of headache, vomiting, and cough. During her evaluation, clinicians incidentally noted elevated blood pressure readings, prompting a referral to our centre for further investigation of hypertension. Approximately four months prior to her presentation, the patient’s mother measured her daughter’s blood pressure and recorded a high reading; however, she did not pursue medical consultation at that time.

**Fig. 2:**
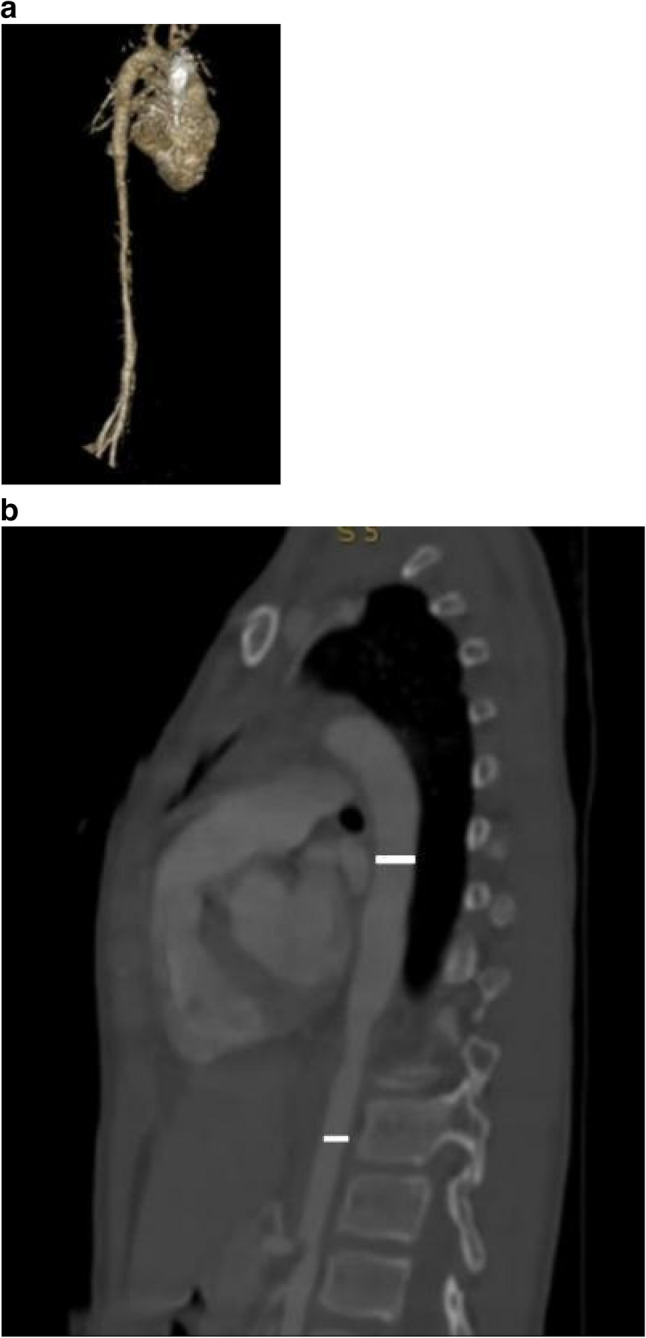
**A** The sagittal view revealed significant segmental narrowing (arrowhead) in the abdominal aorta. **B** Sagittal view: long aortic narrowing from the diaphragm, with thoracic-abdominal size mismatch (lines)

The patient appeared healthy, alert, and active, with no signs of respiratory distress. Vital signs were largely within normal limits, except for elevated blood pressure. The growth parameters included a weight of 31 kg (50th percentile), a height of 132 cm (25th percentile), and a body mass index of 12.9. A comprehensive four-limb blood pressure assessment revealed readings of 125/81 mmHg in the right arm, 122/82 mmHg in the left arm, 123/85 mmHg in the right leg, and 110/70 mmHg in the left leg. There were no significant differences between the arm and leg blood pressure measurements, with the overall blood pressure recorded at the 95th percentile (113/75 mmHg). Cardiovascular examination was generally normal, except for a systolic ejection murmur. The femoral pulses were palpable, and the radiofemoral delay was absent. Systemic examinations, including ocular, thoracic, and abdominal evaluations, were unremarkable. The initial laboratory evaluations included a complete blood count, a peripheral smear, urea and electrolytes, liver function tests, vitamin D levels, parathyroid hormone assessment, and thyroid function tests. Fresh morning samples were also used for urinalysis, urine culture and sensitivity testing, urine protein quantification, urine creatinine measurement, and urine albumin-to-creatinine ratio analysis. The laboratory results were largely unremarkable. An echocardiogram revealed normal intracardiac structures, except for mild left ventricular hypertrophy. While Doppler of the abdominal aorta showed the same signs as in the first patient, who was characterised by diastolic runoff; a CT angiogram of the thoracic and abdominal aorta was recommended. Various imaging studies, including abdominal ultrasound and Doppler assessments of the renal, mesenteric, and lower limb vessels, have yielded unremarkable results. The CT angiogram revealed left ventricular hypertrophy and smooth narrowing of the abdominal aorta, suggesting MAS (Fig. 2a and b).

Furthermore, the assessments of blood glucose levels, antistreptolysin antibodies, complement levels, lipid profiles, cholesterol levels, erythrocyte sedimentation rates, and C-reactive protein, immunoglobulin, renin, and aldosterone levels yielded no significant findings. The patient was prescribed a variety of antihypertensive medications, including hydralazine, amlodipine, and enalapril. A referral was subsequently made to a higher specialised centre for further evaluation and management.

## Discussion

MAS typically develops in children and adolescents due to genetic factors, such as Alagille syndrome and Williams syndrome, or acquired conditions, including Takayasu arteritis and intrauterine infections. The clinical manifestations of MAS vary depending on the extent and location of arterial narrowing [3]. The patients in this case study were two 10-year-old Saudi females who had previously been healthy and presented with elevated blood pressure readings. However, the primary complaints were distinct in each case. The first patient experienced dizziness and transient loss of consciousness, whereas the second patient reported headache, vomiting, and cough. Despite both individuals exhibiting hypertension, the severity of their conditions varied. The first patient presented with severe hypertension accompanied by a significant blood pressure gradient between the arms and legs. In contrast, the second patient had lower blood pressure readings, with no observable gradient between the upper and lower extremities.

During the physical examination, the first patient presented with clinical signs of coarctation of the aorta, including a bounding radial pulse and diminished lower-limb pulses. Conversely, the second patient presented with normal pulses and no detected blood pressure gradient between the arms and legs. However, both patients had an ejection systolic murmur.

The suspicion of MAS in the first patient was based on the observed difference in blood pressure between the four limbs and the diminutive abdominal aorta, as noted in the echocardiographic assessment, which also revealed abnormal diastolic flow. While the second patient did not show a significant gradient in arm-leg BP or diminished femoral pulse, MAS was suspected due to refractory hypertension, mild left ventricular hypertrophy and the abnormal holodiastolic flow in the abdominal segment of the aorta on echocardiography. In some patients with moderate MAS, classic findings may not be present, making imaging essential for diagnosis.

Hence, slight echocardiographic abnormalities can serve as important clues to the diagnosis of rare cardiovascular anomalies. Also, previous imaging-based studies have proved the role of multimodality imaging in detecting uncommon cardiovascular anatomical diseases that may not be noticed during physical examination. For instance, Kula et al. discussed the usefulness of advanced radiological techniques in diagnosing these anomalies, emphasising the need for a high index of suspicion when encountering unusual findings [4]. In both instances, abnormal diastolic flow on echocardiography necessitated further investigation with CTA. The first patient presented with bilateral proximal renal artery stenosis, evident on renal artery pulsus parvus et tardus waveforms, whereas the second patient presented with normal findings on renal Doppler assessment. In the initial case, the patient’s hypertension was refractory to pharmacologic treatment. Conversely, in the second patient, hypertension was initially resistant to hydralazine but showed partial improvement with the use of antihypertensive medications.

Although cardiovascular abnormalities constitute a primary cause of morbidity and mortality in Williams–Beuren syndrome (WBS), the occurrence of MAS within this syndrome is relatively uncommon. Byoun et al. reported a case involving an asymptomatic 17-year-old male with systemic hypertension who was ultimately diagnosed with WBS and MAS. Like the first case presented here, this patient did not exhibit the classic features of WBS, such as characteristic facial dysmorphism and short stature. In contrast to our patient, whose genetic testing yielded inconclusive results, the case reported by Byoun et al. involved a mutation characterised by an atypical small deletion at 7q11.23, alongside a benign variant that duplicated Yq11.223.Furthermore, the stenosis in Byoun et al.’s case was diffusely located within the thoracoabdominal aorta. It did not impact the renal arteries or other major vessels, distinguishing it from the first case, which exhibited more severe stenosis involving the renal arteries [5]. Although transthoracic echocardiography (TTE) has a restricted ability to detect MAS, Wang et al. published a case of a 14-year-old female with dizziness and refractory hypertension that was discovered via TTE [6]. In both of our patients, the observed abnormal diastolic flow pattern in the descending thoracic aorta was a significant indicator, warranting further assessment with CT angiography.

In 2007, Sohn et al. reported a case involving a 14-year-old female patient from Iraq who underwent transthoracic echocardiography, which revealed the presence of LVH. This finding underscores the importance of echocardiographic assessment in evaluating cardiac conditions and in identifying patients with significant aortic pathology [7]. Although Sohn et al. reported an Arabian female with a similar condition, this case report is the first article in Saudi Arabia to report mid-aortic syndrome.

The treatment of MAS requires a multidisciplinary approach, including HTN specialists, nephrologists, cardiologists, interventional radiologists, and vascular surgeons. The management strategy should initially focus on lowering BP, with surgical intervention considered based on the degree of vascular involvement. The intervention can be balloon angioplasty, bypass grafting, stent implantation, renal artery reconstruction or patch aortoplasty. Careful assessment of the patient’s case is necessary, as the method of intervention and its timing are determined by age, HTN severity, anatomy, vascular lesions, and organ involvement. Both of our patients were referred to a tertiary hospital for further evaluation and long-term care. Our study did not include follow-up on outcomes; rather, it focused on the diagnostic process of the disease, which may present with various nonspecific manifestations in addition to elevated blood pressure. It is a rare aetiology of resistant hypertension and is characterised by only subtle indications observable on TTE.

## Conclusion

This case report underscores the importance of diagnostic evaluation in mid-aortic syndrome. It emphasises the role of transthoracic echocardiography in detecting subtle indicators critical to the diagnosis of this condition. Mid-aortic syndrome may be considered in any child or adolescent presenting with refractory hypertension, irrespective of their symptoms, or in cases where hypertension has been identified incidentally. A thorough physical examination for signs of aortic coarctation is warranted, as it may provide preliminary indications; however, this examination may yield inconclusive results, particularly in mild cases where physical signs are absent. An echocardiogram may provide important clues in diagnosing mid-aortic syndrome.

## Supplementary Information

Supplementary Material 1.

## Data Availability

No datasets were generated or analysed during the current study.

## Abbreviations

CoA: Coarctation of the aorta
MAS: Mid-aortic syndrome
HTN: Hypertension
BP: Blood pressure
LVH: left ventricular hypertrophy
CTA: Computerised Tomographic Angiography
WBS: Williams–Beuren Syndrome Scale
TTE: Transthoracic echocardiography
LIMA: Left Internal Mammary Artery
