# Supplementary material for: Mid-aortic syndrome in two Saudi children with refractory hypertension: a case report
Source: Egypt Heart J. 2026 Jun 24;78:50. doi: 10.1186/s43044-026-00758-z (PMC13294414; doi:10.1186/s43044-026-00758-z)
Supplement: Supplementary file 1 — Supplementary Material 1. [file 43044_2026_758_MOESM1_ESM.pdf]

CARE
case report guidelines

CARE Checklist of information to include when writing a case report

BY-NC-ND

Topic	Item	Checklist item description	Reported on Line
Title	1	The diagnosis or intervention of primary focus followed by the words "case report"	1
Key Words	2	2 to 5 key words that identify diagnoses or interventions in this case report, including "case report"	16
Abstract (no references)	3a	Introduction: What is unique about this case and what does it add to the scientific literature?	4-1
	3b	Main symptoms and/or important clinical findings	5-6
	3c	The main diagnoses, therapeutic interventions, and outcomes	5-10
	3d	Conclusion—What is the main "take-away" lesson(s) from this case?	11-15
Introduction	4	One or two paragraphs summarizing why this case is unique (**may include references**)	11-16
Patient Information	5a	De-identified patient specific information.	34-67
	5b	Primary concerns and symptoms of the patient.	34-36 , 67-71
	5c	Medical, family, and psycho-social history including relevant genetic information	34, 67
	5d	Relevant past interventions with outcomes	73-72,38-37
Clinical Findings	6	Describe significant physical examination (PE) and important clinical findings.	39-50,74-83
Timeline	7	Historical and current information from this episode of care organized as a timeline	39-50,74-83,38-57
Diagnostic Assessment	8a	Diagnostic testing (such as PE, laboratory testing, imaging, surveys).	51-60,84-98
	8b	Diagnostic challenges (such as access to testing, financial, or cultural)	129-131,144-147
	8c	Diagnosis (including other diagnoses considered)	123-128
	8d	Prognosis (such as staging in oncology) where applicable	22-24
Therapeutic Intervention	9a	Types of therapeutic intervention (such as pharmacologic, surgical, preventive, self-care)	102-100,66
	9b	Administration of therapeutic intervention (such as dosage, strength, duration)	102-100
	9c	Changes in therapeutic intervention (with rationale)	133-131
Follow-up and Outcomes	10a	Clinician and patient-assessed outcomes (if available)	158-157
	10b	Important follow-up diagnostic and other test results	94-98,60-57
	10c	Intervention adherence and tolerability (How was this assessed?)	133-132
	10d	Adverse and unanticipated events	133-132
Discussion	11a	A scientific discussion of the strengths AND limitations associated with this case report	157-164
	11b	Discussion of the relevant medical literature **with references**.	134-156
	11c	The scientific rationale for any conclusions (including assessment of possible causes)	121-143
	11d	The primary "take-away" lessons of this case report (without references) in a one paragraph conclusion	165-173
Patient Perspective	12	The patient should share their perspective in one to two paragraphs on the treatment(s) they received	
Informed Consent	13	Did the patient give informed consent? Please provide if requested 179-184	Yes ☐ No ☐
